# *In Vitro* Comparison of Pushout Bond Strength of ProRoot MTA, Biodentine and TheraCal

**DOI:** 10.4317/jced.58893

**Published:** 2021-12-01

**Authors:** Neda-Kheirkhah Dabbagh, Ehsan Esnaashari, Hengameh Bakhtiar, Mohammad-Hosein Nekoofar, Milad Ghezelsofla

**Affiliations:** 1 Postgraduate Student, Department of restorative dentistry, School of dentistry, Shahid Beheshti University of medical sciences, Tehran, Iran; 2 Assistant Professor , Department of Endodontics, Dental Material Research Center, Faculty of Dentistry , Tehran Medical Sciences, Islamic Azad University, Tehran, Iran; 3 Associate Professor , Department of Endodontics, Dental Material Research Center, Faculty of Dentistry , Tehran Medical Sciences, Islamic Azad University, Tehran, Iran; 4 Associate Professor, Department of Endodontics, School of Dentistry, Tehran University of Medical Sciences, Tehran, Iran; 5 PHD student, Department of Tissue Engineering,Central tehran Branch,Islamic Azad University,Tehran, Iran

## Abstract

**Background:**

One problem encountered in vital pulp therapy is the dislodgment of biomaterial as the result of the application of mechanical condensation forces for the final restoration of the cavity or occlusal loads.

**Material and Methods:**

In this *in vitro*, experimental study, 90 dentin discs were prepared with Gates Glidden drills to have a 1.3 mm canal diameter. The specimens were divided into nine groups (n=10). ProRoot MTA, Biodentine, and TheraCal were applied in groups 1-3, 4-6, and 7-9, respectively. The PBS was measured after 15 minutes, four hours, and three days and mode of failure was determined.

**Results:**

The interaction effect of time and material on PBS was statistically significant (*P*<0.003). The PBS of Biodentine and ProRoot MTA significantly increased over time (*P*=0.000). At 15 minutes and four hours, the PBS of TheraCal was higher than that of Biodentine and ProRoot MTA (*P*=0.000). Our results showed the predominant type of bond failures in Biodentine and Theracal groups was cohesive, whereas it was adhesive for ProRootMTA.

**Conclusions:**

Theracal showed higher values of bond strength than Biodentine and ProRootMTA at 15 minutes & four hours and may thus be better options for single session of VPT.

** Key words:**Biodentine, Mineral Trioxide Aggregate, Pushout Bond Strength, TheraCal.

## Introduction

One problem encountered in vital pulp therapy is the dislodgment of biomaterial as the result of the application of mechanical condensation forces for the final restoration of the cavity or occlusal loads (1,2). Not paying attention to this issue may result in dislodgement of biomaterial and loss of seal leading to bacterial leakage and eventual failure of vital pulp therapy (1,3,4). Restorative materials must be biocompatible, prevent leakage, have an optimal adaptation to the tooth structure, and possess high pushout bond strength (PBS) against dislodging forces (such as the force applied during packing of final restorative material or occlusal loads) especially for the treatment of perforations (5). Several materials have been used for vital pulp therapy such as gold, intermediate restorative material, calcium silicate cement (CSC), and mineral trioxide aggregate (MTA) (2,6).

MTA has high PBS and elicits optimal tissue response. However, its long setting time, difficult handling, high cost, and risk of tooth discoloration are among its drawbacks (2,7-10). ProRoot MTA (Dentsply Endodontics, Tulsa, OK, USA) mainly contains dicalcium and tricalcium silicate, bismuth oxide, and some other oxides (Table 1) that can set in presence of water (11-13). Some other calcium silicate-based materials were introduced to overcome the shortcomings of MTA (14). For example, Biodentine (Septodont, Saint Maur des Fosses, France; Table 1) has comparable traits to MTA which make it a proper substitute. Its handling is even much easier and it setting time is shorter than those of MTA suggesting it as a better alternative (2,11,13-16). Another novel alternative is a light-curing Resin Modified Calcium Silicate (RMCS) material called TheraCal (Bisco Inc., Schaumburg, IL, USA; Table 1) that is low soluble with physical advantages that make it a proper option for protecting the dental pulp complex and being applied as barrier (13). This RMCS material has preponderance over MTA in terms of sealing ability, low solubility, and low interfacial micro-leakage. TheraCal also release more calcium ions compared with MTA resulting in further formation of apatite and secondary dentin. It has been designed for direct and indirect pulp capping (14,16). It contains 45% minerals, 5% radiopaque materials, 5% hydrophilic materials (fumed silica) and about 45% resin (13,16). It forms an accepTable physicochemical bond to dentin and has the optimal sealing ability (13).

**Table 1:**
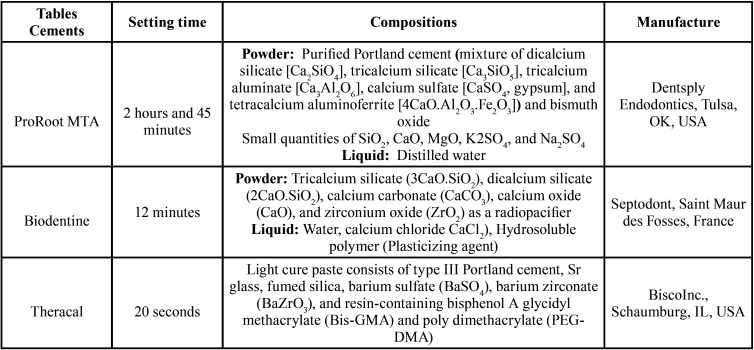
Composition of calcium silicate-based cements.

Considering the significance of achieving adequate PBS in biomaterials prior to the final restoration of a tooth in case of vital pulp therapy or treatment of restorations, this study aimed to compare the PBS of ProRoot MTA, Biodentine, and TheraCal at 15 minutes, four hours, and three days after their application.

## Material and Methods

-Teeth selection:

This *in vitro* experimental study was conducted on 90 dentin discs cut out of single-rooted human canine teeth, extracted for periodontal reasons. The selected teeth did not have cracks or carious lesions, had not undergone endodontic treatment, did not have internal or external root resorption, and did not have calcified canals (ensured radiographically). The teeth were selected using convenience sampling and were immersed in 0.5% chloramine T solution for one week for disinfection.

-Preparation of specimens for push-out bond strength (PBS) test:

The teeth were then decoronated at the cemento-enamel junction and mounted in acrylic resin. Transverse sections were made at the midpoint of the root length by a low-speed diamond saw underwater coolant to obtain discs with 1 mm thickness. The canal of specimens was prepared using #2-5 Gates Glidden drills to have 1.3 mm diameter. Canals were irrigated with 5mL 2.5% NaOCl + 9% Dual Rinse HEDP for 1 minute after mechanical preparation for removing smear layer.

-Sample Grouping :

The specimens were then randomly divided into nine groups (n=10) using block randomization. After ensuring that the specimens did not have cracks, ProRoot MTA, Biodentine, and TheraCal were applied into the canal of the specimens in groups 1-3, 4-6, and 7-9, respectively in the form of a thin coat. Excess material was trimmed by a scalpel. The specimens were wrapped in moist gauze and incubated at 37°C and 100% humidity. The three groups for each biomaterial were designed for the assessment of PBS at 15 minutes, four hours, and three days.

15 minutes: Because of setting time of TheraCal and Biodentine.

Four hours: Because of setting time of ProRoot MTA.

Three days: To assess the effect of time on PBS.

**Figure 1:**
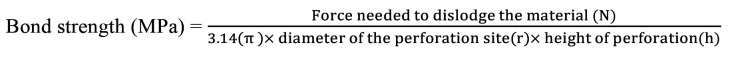
Formula.

-PBS test:

The push-out test was conducted using a universal testing machine (Z050, Zwick/Roell, Ulm, Germany). the specimens were placed on a metal slab with a hole at the center for free movement of the piston. The compressive load was applied in the apicocoronal direction at a crosshead speed of 1 mm/min using stainless steel plungers of 0.6 mm positioned so that they contacted only the filling material. The maximum force (F) applied at bond failure was recorded in newtons The pushout bond strength was calculated in MPa using the following formula: (Fig. 1).

-Failure mode analysis:

The mode of failure was determined under a stereomicroscope (Nikon, Tokyo, Japan) at x10 magnification. Each sample was classified into one of the following failure modes: ‘adhesive failure’ at the biomaterial-dentin interface, ‘cohesive failure’ within the biomaterial or dentin, or ‘mixed failure’ in both types.

-Statistical analysis :

Data were analyzed using SPSS version 22 (SPSS Inc., IL, USA) via descriptive (mean and standard deviation) and analytical statistics. The normality of the PBS data was assessed using the Kolmogorov-Smirnov test. Since the data were found to have a normal distribution, two-way ANOVA, one-way ANOVA, and posthoc Tukey’s test were applied to assess the effect of type of biomaterial (ProRoot MTA, Biodentine and TheraCal) and time (15 minutes, four hours, and three days) on the PBS. *P*<0.05 was considered statistically significant.

## Results

Table 2 shows the mean PBS in the three groups of ProRoot MTA, Biodentine, and TheraCal at different time points.

**Table 2:**
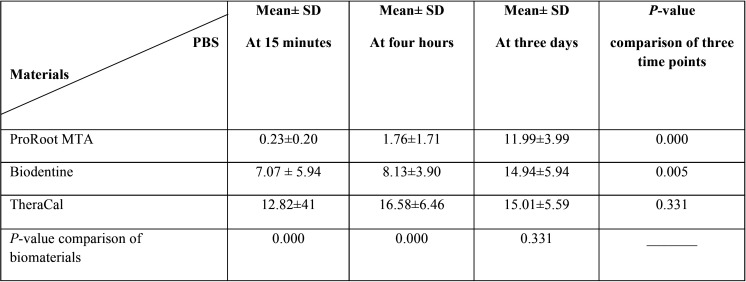
Mean PBS of ProRoot MTA, Biodentine and TheraCal at different time point.

Two-way ANOVA showed that the interaction effect of time and biomaterial on PBS was statistically significant (*P*=0.003). Thus, one-way ANOVA and then Tukey’s post hoc test was applied to separately assess the effect of time and type of biomaterial on PBS.

Regarding the effect of time, the three groups were significantly different in terms of PBS at 15 minutes (*P*=0.000). Pairwise comparisons were then carried out and the results showed significant differences between Biodentine and ProRoot MTA (*P*<0.034), and TheraCal and Biodentine (*P*=0.010) in terms of PBS at 15 minutes.

At four hours, the three groups were significantly different in terms of PBS (*P*=0.000). Pairwise comparisons revealed significant differences between TheraCal and Biodentine (*P*<0.001), TheraCal and ProRoot MTA (*P*<0.000), and Biodentine and ProRoot MTA (*P*<0.010) in terms of PBS at four hours.

At three days, the three groups were not significantly different in terms of PBS (*P*>0.05), (Table 3).

**Table 3:**
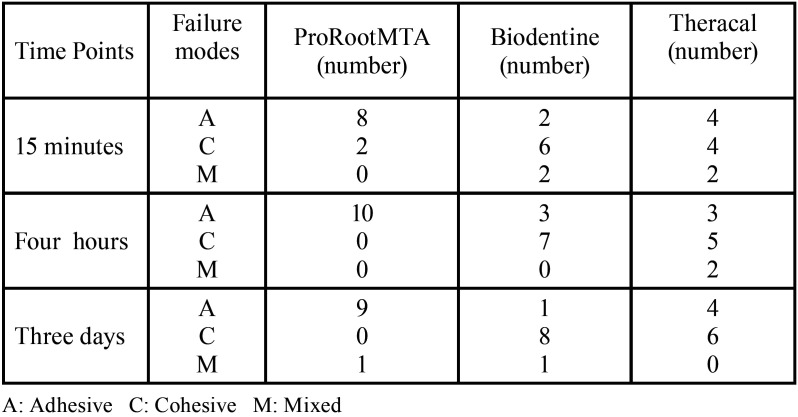
Frequency distribution of modes of failure in the three groups at different time points.

Regarding the effect of type of biomaterial, no significant difference was noted in PBS of TheraCal at 15 minutes, four hours, and three days (*P*>0.05). The difference in PBS of Biodentine was significant at different time points (*P*=0.005). Thus, pairwise comparisons were performed, which showed significant differences in PBS of Biodentine between 15 minutes and three days (*P*=0.005) and four hours and three days (*P*=0.036) but the difference in PBS of Biodentine at 15 minutes and four hours was not significant (*P*>0.05). For ProRoot MTA, significant differences were noted in PBS among the three-time points (*P*=0.000). Pairwise comparisons showed significant differences in PBS of ProRoot MTA between 15 minutes and three days (*P*=0.000) and four hours and three days (*P*=0.000) but the difference between 15 minutes and four hours was not significant (*P*>0.05).

## Discussion

After vital pulp therapy, the success of treatment depends on the proper restoration of the cavity. However, before placing the final restoration, the biomaterial must gain adequately high PBS to be able to well resist the condensing forces applied to the final restorative material. The condensing force for amalgam can be as high as 8.9±2.4 MPa and 5.5±1.8 MPa by the use of small and large condensers, respectively (17). Such a high force can result in dislodgement of biomaterial used for vital pulp therapy (3,18). Thus, the PBS of biomaterial used for vital pulp therapy is highly important in the clinical setting (3).

 According to former reports, the most dependable test that assesses the materials’ resistancy against dislodgement forces is the POBS test (2,19). Calcium silicate cement or CSC is a biocompatible material that can properly promote calcium-phosphate precipitation at its interface with dentin, then, it is highly recommended for vital pulp therapy (8). This material releases a good amount of bioavailable calcium (Ca) ions that can activate ATP and elevate the expression of bone-associated proteins resulting in augmentation of the mineralization process (20). The biomineralization process lead in forming an interfacial layer as well as tag-like structures that make calcium silicatebased materials highly resistant to displacing from the dentine interface (21).

In instrumented canal walls, using root canal irrigant (RCI) materials is necessary in order to create a tight seal after exposing the dentinal tubules since during the mechanical preparation, the bond strength reduces due to forming a smear layer in the root canal system. One of the most extensively used RCI materials is sodium hypochlorite (NaOCl) that owns good antimicrobial and tissue-dissolving properties (2,22). The effects of RCI materials can be improved in association with chelator materials such as Etidronic acid (1-hydroxyethane 1,1- bisphosphonate or HEBP) (2). Research has also shown that the combination of NaOCl and HEBP maintains the properties of both materials and has no negative impact on the CSC’s PBS (2).

Keeping samples in gauze impregnated with normal saline for 48 hours before PBS test is recommended for moistening of CSC which provides a greater comprehensive strength for the cement, improving the retention characteristics, and increasing the push-out strength of CSC (23). Bozeman *et al*. simulated the clinical setting by wrapping the root sections in a gauze dipped in the synthetic tissue fluid (24). However, we used a gauze dipped in water since the synthetic tissue fluid may interfere with intratubular mineralization and affect the PBS.

This study assessed the PBS of ProRoot MTA, Biodentine, and TheraCal at 15 minutes, four hours, and three days after their application and showed that the PBS of Biodentine and ProRoot MTA at 15 minutes and four hours was less than the condensing forces commonly applied for condensation of amalgam into the cavity. Thus, more than four hours is required to achieve ultimate strength. At three days, the PBS of Biodentine and ProRoot MTA significantly increased and their bond strength exceeded the condensing forces of amalgam. TheraCal, however, showed bond strength equal to or higher than the condensing forces of amalgam at 15 minutes, four hours, and three days. This shows that TheraCal reached its ultimate strength within 15 minutes after its application. This indicates that TheraCal can be used inside the canal and the cavity can be permanently restored immediately within the same session without requiring temporary filling material. Whereas, MTA shows a less initial compression strength & PBS and increasing its value required time and moisture (14,25).

Biodentine is supplied both as liquid and capsulated powder. The composition Biodentine powder is modulated so that its physical properties and usability are improved. Compared with MTA, setting accelerators and softeners are added to the Biodentine formulation that have made its setting faster and the bacterial contamination probability is decreased by inhibiting the prolonged leakage (10,16).

 Another light-cured, resin-modified calcium silicate is TheraCal that also contains zirconium oxide which can be directly placed on the operative site without prior mixing and handling procedures. However, the manufacturer has instructed this novel light-curable MTA-like material for less than 1-mm increments, it was shown in a study that TheraCal has the capability of being cured to the depth of 1.7 mm (16). Accordingly, the initial strength of this lightcuring material is immediately gained after application. TheraCal also forms more sTable crystals with less microporosity and microhardness values that make it minimally sensitive to the environment acidity (7). Also, TheraCal is a proper cement for either direct or indirect pulp-capping since it produces minimal heat during polymerization that minimizes the probable adverse effects on the pulp (26). Additionally, the setting mechanism and calcium releasing properties of TheraCal LC is modified using a hydrophilic monomer that causes TheraCal LC acting as a scaffold for dentin regeneration (13,27). TheraCal LC absorbs the dentinal fluids and release of calcium and hydroxide ions raising the apatite formation as the respond of the tooth which confers a natural sealing property to the material and helps secondary dentin bridge formation (13). The resin nature of TheraCal LC augments its compressive strength and bond strength to that confirms it as a beneficial pulp capping material. TheraCal LC is a promising pulp capping material since it can shorten the time and cost for both patients and dentists since allow positing the final restoration on the in the same visit (25). These results are in accordance with those of Alsubait *et al*. and Rahimi *et al*. who reported no significant differences in the bond strength of Biodentine and MTA after three days (28,29).

Aggarwal *et al*. compared the PBS of ProRoot MTA and Biodentine for furcal perforation repair at 24 hours and seven days and showed that at 24 hours, the PBS of ProRoot MTA was significantly lower than that of Biodentine (30). But over time, its PBS increased, which was in line with our results. Gasperi *et al*. reported push-out bond strength of Theracal to dentin was satisfactory and significantly higher than that of MTA after 24 hours (25). Aggarwal *et al*. and Nagas *et al*. showed that the PBS of Biodentine was higher than that of ProRoot MTA, which was different from our results at three days (30,31).

In the current study, the mode of failure was also determined by inspection of specimens under a stereomicroscope at 10X magnification. Different modes of fracture can be explained by the difference in the size of particles of different biomaterials, affecting the penetration of cement into dentinal tubules. Smaller particles and homogenous elements better penetrate dentin and cause mechanical interlocking, resulting in eventual cohesive failure of cement (21). Those authors attributed this failure mode with the material dislodgement due to its high compressive strength. Our results showing the bond failures predominantly in Biodentine and Theracal groups (cohesive type) as well as ProRootMTA groups (adhesive type) was in agreement with the findings of Shokouhinejad *et al*. (22). Therefore, we conclude that applying TheraCal LC should be associated with caution due to its biological and chemical properties such as resinous nature. Another possible problematic instance is the presence of residual monomers that are suggested to be associated with the inflammatory consequences that cause a low desirable response in the pulps. As a consequence, the inadequate clinical and experimental evidence about TheraCal LC necessitates further investigation in order to deduce a comprehensive and reliable judgment about this material from a biological point of view.

## Conclusions

In vital pulp therapy with ProRoot MTA and Biodentine, more than four hours is required for the final set of material and thus, final restoration must be postponed to the next session ideally three days after the first session. However, in the use of TheraCal, the final restoration can be placed as soon as 15 minutes after its application and the treatment can be accomplished within a single session.
